# Long-Term Postdeployment Clinical Subtypes of Risk and Resiliency in Brain Injury and Neurodegeneration

**DOI:** 10.1001/jamanetworkopen.2025.47954

**Published:** 2025-12-10

**Authors:** Christine L. Mac Donald, Jason Barber, David Hunt, Jalal B. Andre, Jesse R. Fann, Kristen Dams-O’Connor, Nancy R. Temkin

**Affiliations:** 1 Department of Neurological Surgery, University of Washington, Seattle; 2 Department of Radiology, University of Washington, Seattle; 3 Department of Psychiatry and Behavioral Sciences, University of Washington, Seattle; 4 Department of Rehabilitation and Human Performance, Icahn School of Medicine, Mount Sinai, New York, New York; 5 Department of Neurology, Icahn School of Medicine, Mount Sinai, New York, New York

## Abstract

**Importance:**

While research has focused on veterans after deployment, fewer studies have examined longitudinal outcomes associated with neurodegeneration. Questions remain about how these trajectories may connect and inform overall risk for later-life neurodegeneration and possible dementia.

**Objective:**

To analyze acute, 1-year, 5-year, and 10-year longitudinal outcomes and magnetic resonance imaging (MRI) data from US military personnel to understand profiles of neurodegenerative resilience and risk.

**Design, Setting, and Participants:**

This prospective longitudinal, cohort study included data from 4 combat-deployed groups: 2 control groups without head injury (36 individuals with blast exposure and 94 individuals without) and 2 groups with combat-related mild traumatic brain injury (TBI) from blast (137 individuals) or blunt trauma (21 individuals). Enrollment occurred from 2008 to 2013 in Afghanistan or following medical evacuation to Landstuhl, Germany, with 1-year, 5-year, and 10-year follow-ups completed in the US. Data were analyzed from October 2024 to March 2025.

**Exposures:**

All participants experienced combat deployment and some participants experienced mild TBI.

**Main Outcomes and Measures:**

Clinical outcomes included 34 measures of neurobehavioral, psychological, and cognitive function completed at 1-year, 5-year, and 10-year follow-ups and high-resolution structural MRI completed at baseline and at the 3 follow-up evaluations. Ten-year post-deployment clinical outcome subtypes were derived from the 34 measures using *k*-means clustering.

**Results:**

A total of 288 service members (mean [SD] age at final follow-up, 41.5 [8.1] years; 263 [91%] male) were assessed. Clustering optimization identified 4 clusters: 1 multidomain resilient to cognitive dysfunction, neurobehavioral and psychological symptoms (124 individuals); 1 resilient to cognitive dysfunction but with elevated neurobehavioral and psychological symptoms (122 individuals); 1 with severe cognitive dysfunction and moderately elevated neurobehavioral and psychological symptoms (14 individuals); and 1 with moderate cognitive dysfunction and severe neurobehavioral and psychological symptoms (28 individuals). Participants in the resilient group were largely nonblast controls, but 45 individuals (36%) had experienced blast mild TBI. Comparing the clusters over time revealed they were significantly different. Significant brain volume differences by cluster were observed comparing them at each time point. The cluster with severe cognitive dysfunction and moderately elevated neurobehavioral and psychological symptoms had the lowest brain volume, particularly in the cerebellum and brainstem.

**Conclusions and Relevance:**

In this cohort study of service members, clinical outcome clustering revealed key postdeployment subtypes that evolved over time. Cerebellar and brainstem volumes were significantly different by cluster. Regional differences were evident years before when stratified by these 10-year clusters. These findings underscore the need to appreciate not only the heterogeneity of brain injury but the heterogeneity of these mild TBI outcomes.

## Key Points

**Question:**

Are combat deployment exposure and mild brain injury associated with later-life neurodegeneration?

**Findings:**

In this cohort study of 288 US military service members, 4 key outcome clusters of resilience and risk were identified also associated with brain volumetric differences. While individuals with mild TBI were enriched in the risk clusters, a notable number were found in the resilient cluster, with comparisons of these same clusters over 1-year, 5-year, and 10-year follow-ups revealing an evolution over time in a clinically meaningful way.

**Meaning:**

These findings underscore the need to better appreciate not only the heterogeneity of brain injury but the heterogeneity of these mild TBI outcomes.

## Teaser

This cohort study examines acute, 1-year, 5-year, and 10-year longitudinal outcomes and magnetic resonance imaging data from US military personnel to understand profiles of neurodegenerative resilience and risk.

## Introduction

While much research has focused on veterans after deployment, fewer studies have examined longitudinal outcomes^1,2,3,4^ and even fewer multiyear postdeployment outcomes.^5^ Questions remain about how these trajectories may connect to and inform the overall risk for later-life neurodegeneration and possible dementia. Exposures sustained in combat, such as mild traumatic brain injury (TBI), have been postulated to further increase this risk beyond deployment exposure itself. In fact, older US military veterans of conflicts after September 11, 2001, with mild TBI have a 2-fold increase dementia risk vs veterans without mild TBI.^6,7^ There is an urgent need to better understand the long-term consequences, as many of these veterans are now reaching the age of greatest dementia risk. The few longitudinal studies that have collected brain magnetic resonance imaging (MRI) data in this military population have yielded mixed results. Some report no volumetric changes,^8^ while others report possible progression of white matter lesioning,^9^ cortical volume reductions,^10^ and possible changes in white matter microstructure^11^ over varying times after deployment.

More recently, the neurodegenerative disease research community has postulated there may be subtypes or endophenotypes to TBI^12,13^ outcomes and conditions, like Alzheimer disease.^14,15^ With this in mind, we sought to understand (1) whether there are key clinical postdeployment subtypes that inform who is at greatest risk of later neurodegeneration and (2) whether mild TBI is associated with poor postdeployment outcome subtypes, which could inform how these exposures relate to previously reported dementia risk.^6,7^ We leveraged the longitudinal clinical outcome and neuroimaging data collected as part of the Evaluation of Longitudinal Outcomes in Mild TBI Active-Duty Military and Veterans (EVOLVE) study^16,17,18^ that uniquely followed service members with and without mild TBI from combat to long-term outcomes. The objective was to analyze the acute, 1-year, 5-year, and 10-year longitudinal outcomes and MRI data from the EVOLVE study to understand profiles of resilience and risk.

### Methods

This cohort study was approved by the University of Washington institutional review board with additional approval from the US Army Medical Research and Materiel Command institutional review board and carried out in accordance with the approved protocol. Reconsent for each follow-up evaluation was provided by all participants according to the Declaration of Helsinki. This study is reported following the Strengthening the Reporting of Observational Studies in Epidemiology (STROBE) reporting guideline.

This analysis used our prospective, observational, longitudinal, multicohort study of deployed service members. Originally enrolled from 2008 to 2013 in combat or following medical evacuation to Landstuhl, Germany, the EVOLVE study^16,17,18^ assessed 4 combat-deployed groups: 2 control groups without head injuries (with or without blast exposure), and 2 groups with combat-related mild TBI arising from blast or blunt trauma. TBI diagnosis and blast exposure were determined by medical personnel in the TBI clinics in Afghanistan or Germany. For blast TBI, all available clinical histories indicated blast plus another mechanism of head injury. None experienced isolated blast injury. All individuals with blast and nonblast TBI met the Department of Defense definition for mild, uncomplicated TBI,^19^ defined as Glasgow Coma Scale score 13 to 15, loss of consciousness 0 to 30 minutes, alteration of consciousness less than 24 hours, posttraumatic amnesia less than 24 hours, and no acute intracranial imaging pathology. All combat-deployed controls were clinically evaluated to be free of signs and symptoms of TBI and additionally no history of blast exposure for nonblast controls. Individuals with prior psychiatric and moderate to severe TBI diagnoses were excluded for all groups. Race and ethnicity was self-identified by the service members, and the study used the National Institutes of Health racial and ethnic categories. Collection of race and ethnicity data is a federal reporting requirement.

### Clinical Outcome Measures

Overall clinical evaluations lasted approximately 5 hours; 1 hour for neurobehavioral evaluation and 2 hours each for cognitive testing and psychological evaluation. During the evaluations, participants took their regularly scheduled medications. All examiners underwent standardized training for evaluation consistency and were blinded to other clinical information. Further details are provided in the eMethods in Supplement 1.

### Neurobehavioral Assessment

The neurobehavioral assessment included a structured clinical interview to assess 29 domains of neurobehavioral function (Neurobehavioral Rating Scale-Revised [NRS]^20^; range, 0-87; higher score indicates worse neurobehavioral symptoms), 2 headache interviews capturing frequency and intensity (Migraine Disability Assessment [MIDAS]^21^; range, 0-270; higher score indicates greater headache frequency; and Headache Impact Test [HIT-6]^22^; range, 36-78; higher score indicates greater headache impact), the Neurological Outcome Scale for TBI (NOS-TBI)^23^ assessing focal neurological deficits associated with TBI (range, 0-58; higher score indicates greater focal neurological deficits), and a TBI history intake interview modified from the Brain Injury Screening Questionnaire (BISQ),^24^ to confirm life history of head injury exposure and identify new head injuries sustained since last evaluation (higher score indicates more brain injury exposure). Participants completed the Quality of Life after Brain Injury (QOL)^25^ questionnaire capturing life satisfaction (range, 6-30; higher score indicates better quality of life) and the Patient-Reported Outcomes Measurement Information System Pain (PROMIS)^26^ questionnaire for chronic pain impairment (range, 8-40; higher score indicates greater impact of pain). Global disability was assessed using the Glasgow Outcome Scale Extended (GOSE; range, 1-8; higher score indicates less global disability).^27^ Further details are provided in the eMethods in Supplement 1.

### Psychological Assessment

The psychological evaluation included both clinician-administered structured interviews and patient self-administered questionnaires. The Clinician-Administered Post-Traumatic Stress Disorder (PTSD) Scale for *DSM-IV* (CAPS; range, 0-136; higher score indicates more severe PTSD)^28^ and Montgomery-Asberg Depression Rating Scale (MADRS; range, 0-60; higher score indicates worse depression symptoms)^29^ for depression were administered as structured interviews. The PTSD Checklist-Military (PCL-M)^30^ for PTSD symptoms (range, 17-85; higher score indicates greater PTSD symptoms), Beck Depression Inventory (BDI-II)^31^ for depression symptoms (range, 0-63; higher score indicates higher depression symptoms), Brief Symptom Inventory–Anxiety module (BSI-A) ^32^ for anxiety symptoms (range, 0-24; higher score indicates higher anxiety symptoms), Insomnia Severity Index (ISI)^33^ for sleep difficulty and impact (range, 0-28; higher score indicates worse insomnia), Michigan Alcohol Screening Test (MAST)^34^ for alcohol misuse (range, 0-22; higher score indicates greater alcohol misuse), Connor-Davidson Resilience Scale (CD-RISC)^35^ assessment of resiliency validated in veterans (range, 0-40; higher score indicates greater resiliency),^36^ and Combat Exposures Scale (CES)^37^ a measure of combat intensity (range, 0-41; higher score indicates greater combat exposure) were completed by participants. Further details are provided in the eMethods in Supplement 1.

### Cognitive Assessment

The following cognitive measures were completed at each follow-up wave: Wechsler Test of Adult Reading (WTAR; higher score indicates better baseline intelligence)^38^; Conner’s Continuous Performance Test II (CPT)^39^, specifically omissions, commissions, hit rate, and hit rate block change (reported as *t*-score, where higher indicates worse performance across all CPT measures); California Verbal Learning Test II (CVLT)^40^, specifically long delay (standard score where higher is better performance), intrusions (standard score where lower is better performance), and B vs A (standard score where higher is better performance); Ruff-Light Trail Learning Test (RULIT)^41^, specifically total and delay (reported as *t*-score; higher score indicates better performance); Trails A and Trails B^42^; Controlled Oral Word Association Test (COWA; score scaled by age and education; higher score indicates better performance)^43^; Iowa Gambling Test (IGT; reported as a *t*-score; higher score indicates better performance)^44^; Delis-Kaplan Executive Function System (DKEFS) color-word interference test,^45^ including naming/reading (T1+T2), inhibition (T3) and inhibition/switching (T4) (range, 1-19 for each scaled score; higher score indicates better performance). Fine gross motor function were assessed by Grooved Pegboard Test (GPT; reported in seconds, with higher indicating slower performance)^46^ and the 25-foot walk (reported in seconds, with higher indicating slower performance). Further details are provided in the eMethods in Supplement 1.

### MRI Acquisition and Imaging Processing

At each evaluation wave (enrollment and 1-year, 5-year, and 10-year follow-ups), a brain MRI scan was acquired that included a structural 3-dimensional T1-weighted image (1 × 1 × 1 mm). Postprocessing of the 3-dimensional T1-weighted high resolution structural MPRAGE image was completed using FreeSurfer^47,48^ version 7.1 for volumetric segmentation. In total, 14 subcortical regions, as well as 68 cortical regions per hemisphere, in addition to 168 total brain volume measures were extracted for further analysis. Further details are provided in the eMethods in Supplement 1.

### Statistical Analysis

EVOLVE participants were classified into postdeployment clinical outcome subtypes based on 34 neurobehavioral, psychological, and cognitive measures assessed at 10 years after injury using *k*-means clustering,^49^ an intuitive machine-learning algorithm that partitions a dataset into *k* prespecified clusters based on feature similarity. Each measure was standardized and aligned for consistent directionality in outcomes. The algorithm initializes by randomly specifying *k* centroids in the data space and assigning every data point to its nearest centroid, then updates by redefining the centroids as the mean location of the assigned data points and repeating. This process continues until the centroid positions have reached sufficient stability. Since the *k*-means algorithm requires complete information on all individuals, an expectation-maximization algorithm was used to impute a few scattered missing values in the dataset. While there are several established methods for identifying the optimal value of *k*, such as minimizing the within-cluster variability or maximizing the intracluster variability, none of these methods identified a clear solution for our dataset. Therefore, we evaluated models with 2 to 9 clusters using *R*^2^, cubic clustering criterion, pseudo *F*, and minimum cluster size (eTable in Supplement 1). The 4-cluster solution was selected as the most interpretable balance of fit and parsimony, as fit statistics remained within acceptable ranges, the smallest cluster size (14 individuals) was substantively meaningful, and models with more than 4 clusters fragmented into very small groups that lacked interpretability. Participants were summarized by cluster on demographic and injury characteristics, with intracluster homogeneity assessed for statistical significance using Kruskal-Wallis tests for continuous variables and ordinal variables and Fisher exact test for categorical variables, conservatively adjusting for multiple comparisons using Bonferroni adjustment. Additionally, each cluster was summarized on all 34 measures used in the *k*-means clustering algorithm, reporting the means and applying color-coding based on SD units from the overall sample mean. We also looked at retrospective changes over time within each cluster in the same way, separately for the neurobehavioral and psychological domains and cognitive domains, using linear mixed-effects regression to evaluate whether these changes were statistically significant. Each domain-specific regression model incorporated all relevant measures standardized as *z *scores, modeling cluster and year as fixed effects, individual and measure as crossed random effects, and including a year × cluster interaction effect. Random effects were modeled with unstructured covariance matrices, allowing all variances and covariances to be freely estimated. Linear contrasts were used to estimate and test pairwise cluster differences within each year and between-year differences within each cluster. Omnibus differences across all years and all clusters were tested by refitting each model separately for each cluster and year respectively. Since normality could not be reasonably assumed for some measures, we repeated these analyses using rank-based analogs as a sensitivity analysis.

We assessed 168 volumetric brain regions by cluster at the 10-year follow-up and assessed differences using Kruskal-Wallis tests, reporting only regions that remained statistically significant after correcting for multiple comparisons using a 5% false-discovery rate (FDR) per Benjamini-Hochberg (*m* = 168). In addition, we repeated this approach using the 5-year, 1-year, and enrollment volumetric brain data grouped by cluster as well to assess retrospective brain imaging changes in parallel with the clinical outcomes. The MRI scanner, head coil, and sequences were all the same by evaluation wave, thus we conducted our analysis by time point.

In all analyses, a 2-sided threshold of *P* < .05 was used to define statistical significance. The *k*-means clustering and mixed-effects regression modeling was carried out using SAS version 9.4 statistical software (SAS Institute), and the imputation and volumetric testing was conducted using SPSS version 26 statistical software (IBM). Data were analyzed from October 2024 to March 2025.

## Results

Of 305 service members who completed the 10-year follow up, 288 individuals (mean [SD] age at final follow-up, 41.5 [8.1] years; 263 [91%] male) had complete datasets that were used for cluster analysis, 6 of which required imputation to fill in a total of 14 missing scores. In total, 94 individuals in the nonblast control group, 137 in the blast mild TBI group, 36 in the blast-exposed control group, and 21 in the blunt mild TBI group were included to cluster analysis (Table 1). We have previously observed comparable demographics in those who complete follow up vs those who do not.^50^

**Table 1.** Participant Characteristics by Postdeployment Subtype Cluster

Cluster	Participants, No. (%)				*P* value^e^
	MD-resilient^a^ (n = 124)	Mild risk–NP^b^ (n = 122)	MD risk–Cog^c^ (n = 14)	MD risk–NP^d^ (n = 28)	
Patient group					
Nonblast control	62 (50)	26 (21)	1 (7)	5 (18)	<.001
Blast TBI	45 (36)	66 (54)	8 (57)	18 (64)	
Blast control	13 (10)	18 (15)	3 (21)	2 (7)	
Nonblast TBI	4 (3)	12 (10)	2 (14)	3 (11)	
Age, mean (SD), y	42.1 (8.1)	40.3 (8.0)	44.4 (8.5)	39.2 (7.8)	.03
Sex					
Male	111 (90)	117 (96)	11 (79)	24 (86)	.03
Female	13 (10)	5 (4)	3 (21)	4 (14)	
Race^f^					
African American	11 (9)	17 (14)	4 (29)	4 (15)	.01
Asian or Pacific Islander	4 (3)	2 (2)	1 (7)	0	
Hispanic or Latino	14 (11)	9 (7)	4 (29)	6 (22)	
Mixed race	1 (1)	2 (2)	0	0	
White	93 (76)	91 (75)	5 (36)	17 (63)	
Education, mean (SD), y	16.0 (2.8)	15.1 (2.1)	15.1 (3.0)	14.7 (2.1)	.02
Branch of service^g^					
Army	86 (69)	99 (81)	14 (100)	20 (71)	.01
Air Force	12 (10)	5 (4)	0	1 (4)	
Marine Corps	11 (9)	16 (13)	0	5 (18)	
Navy	15 (12)	2 (2)	0	2 (7)	
Deployments, mean (SD), No.	2.6 (1.7)	2.3 (1.6)	2.5 (1.3)	2.2 (1.5)	.39
Combat Exposure Scale score, mean (SD)^h^	18 (11)	22 (9)	25 (9)	23 (13)	.005
Evacuation status					
Medical	86 (69)	91 (75)	12 (86)	16 (57)	.18
Nonmedical	38 (31)	31 (25)	2 (14)	12 (43)	
Subsequent TBIs, mean (SD), No.	0.11 (0.32)	0.28 (0.61)	0.57 (0.76)	0.46 (0.69)	.001
Service separation at at 10 y eval					
No	39 (31)	16 (13)	2 (14)	4 (14)	.003
Yes	85 (69)	106 (87)	12 (86)	24 (86)	
Moderate-severe PTSD at 10 y					
No	123 (99)	92 (75)	8 (57)	3 (11)	<.001
Yes	1 (1)	30 (25)	6 (43)	25 (89)	
Current disability, mean (SD), %	58 (40)	83 (23)	95 (9)	88 (23)	<.001

Abbreviations: MD, multidomain; PTSD, posttraumatic stress disorder; TBI, traumatic brain injury.

^a^ Classified as individuals resilient to cognitive dysfunction and neurobehavioral and psychological symptoms.

^b^ Classified as individuals resilient to cognitive dysfunction but with elevated neurobehavioral and psychological symptoms.

^c^ Classified as individuals with severe cognitive dysfunction and moderately elevated neurobehavioral and psychological symptoms.

^d^ Classified as individuals with moderate cognitive dysfunction and severe neurobehavioral and psychological symptoms.

^e^ Statistical significance by Kruskal-Wallis and Fisher exact test.

^f^ Mixed race included 2 individuals identifying as Black and White and 1 individual identifying as Asian and White. *P* value assessed with White as the reference group.

^g^ *P* value assessed with Army as the reference group.

^h^ Range, 0 to 41; with higher score indicating greater combat exposure.

Optimization identified 4 clusters: 1 multidomain (MD) resilient to cognitive dysfunction, neurobehavioral and psychological symptoms (MD-resilient; 124 individuals); 1 resilient to cognitive dysfunction but elevated neurobehavioral and psychological symptoms (mild risk–NP; 122 individuals); 1 showing more severe cognitive dysfunction and moderately elevated neurobehavioral and psychological symptoms (14 individuals; MD risk–Cog); and 1 with moderate cognitive dysfunction and substantially elevated neurobehavioral and psychological symptoms (28 individuals; MD risk–NP). Significant differences in demographics were observed across cluster groups, with the proportion of control participants and participants with mild TBI by cluster being the most notably different (Table 1). Analysis of patient profiles revealed that the MD-resilient individuals were more likely to be uninjured deployed service members with higher education (mean [SD], 16.0 [2.8] years) but 45 individuals (36%) had experienced blast mild TBI injury, suggesting a subset of individuals with blast TBI are resilient to these exposures in a clinically meaningful way. In fact, nonblast controls were 4 times more likely to be in MD-resilient. Mild risk–NP was almost 70% service members with blast exposure (66 individuals [54%] with blast mild TBI; 18 control individuals [15%] with blast exposure), but 26 individuals (21%) were nonblast controls. The MD risk–Cog and MD risk–NP clusters largely included individuals with blast exposure, heavily weighted toward blast mild TBI, but did include some nonblast controls. While low, there were significant differences in the number of subsequent head injury exposures in the 10 years after enrollment with higher numbers reported in MD risk–Cog and MD risk–NP clusters (Table 1). There were also significant differences in the percentage who had separated from service by 10-year follow-up), current disability percentage rating, and PTSD burden, with the highest in the MD risk–Cog and MD risk–NP clusters (Table 1). In contrast, no significant differences were observed in number of deployments, age, sex, race, education, or branch of service that held after correction for multiple comparisons.

### Postdeployment Subtypes at the 10-Year Follow-Up

**Figure 1.**
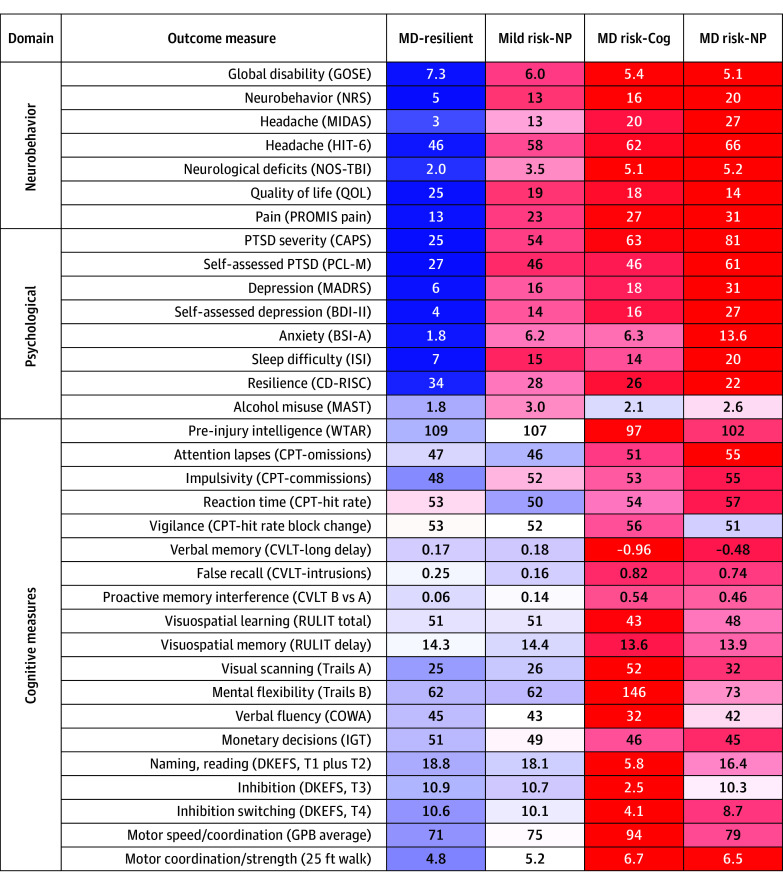
Clinical Outcome Postdeployment Subtypes at 10-Year Follow-Up Values reported are means for each measure by cluster. Color shading indicates the number of SDs from the overall mean, with brighter colors indicating larger deviation. Color shading was done as a continuous gradient representing the magnitude of the standardized effect. Blue shading is better performance while red shading indicates worse performance. MD-resilient was individuals with multidomain (MD) resilience to cognitive dysfunction and neurobehavioral and psychological symptoms; mild risk–NP, resilient to cognitive dysfunction but with elevated neurobehavioral and psychological symptoms; MD risk–Cog, severe cognitive dysfunction and moderately elevated neurobehavioral and psychological symptoms; MD risk–NP, moderate cognitive dysfunction and severe neurobehavioral and psychological symptoms. Measures are described in the Methods.

Comparing risk profiles, there were significant heatmap differences across clusters by mixed-effect regression (Figure 1; eFigure 1 in Supplement 1). As shown in Figure 1, MD-resilient not only performed generally better than the other clusters across all 3 main domains (neurobehavioral, psychological, and cognitive) but was also found to strongly outperform on neurobehavioral and psychological measures. Mild risk–NP had slightly elevated neurobehavioral and psychological symptoms, with cognitive performance within reference ranges. MD risk–Cog had the poorest cognitive performance but notably worse neurobehavioral and psychological symptoms than the MD-resilient and mild risk–NP clusters. All but 2 pairwise cluster differences were statistically significant, and there were 2 pairwise cluster comparisons within each domain where the overall means differed by more than 1 SD. MD risk–NP performed poorly across all domains of function, with particular enrichment for higher neurobehavioral and psychological symptoms. By measure, only the measure of alcohol misuse did not discriminate clusters.

### Longitudinal Evaluation of Postdeployment Subtypes

**Figure 2.**
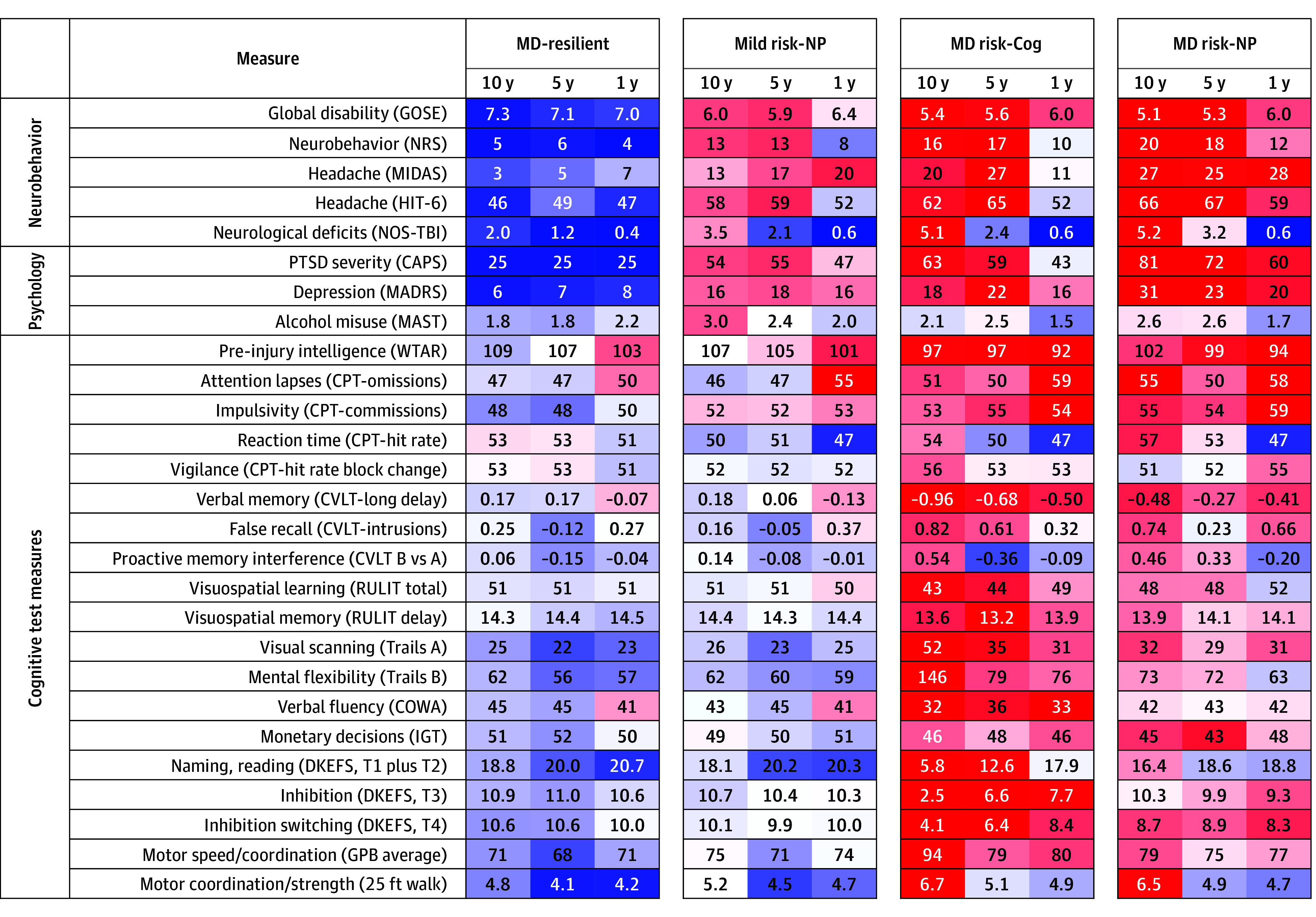
Longitudinal Comparison of Cluster Features: 1-Year, 5-Year, 10-Year Postdeployment by SubType Values reported are means for each measure by cluster. Color shading indicates the number of SDs from the overall mean (based on the 10-year values) with brighter colors indicating larger deviation. Color shading was done as a continuous gradient representing the magnitude of the standardized effect. Blue shading is better performance while red shading indicates worse performance. MD-resilient was individuals with multidomain (MD) resilience to cognitive dysfunction and neurobehavioral and psychological symptoms; mild risk–NP, resilient to cognitive dysfunction but with elevated neurobehavioral and psychological symptoms; MD risk–Cog, severe cognitive dysfunction and moderately elevated neurobehavioral and psychological symptoms; MD risk–NP, moderate cognitive dysfunction and severe neurobehavioral and psychological symptoms. Measures are described in the Methods.

Leveraging the longitudinal EVOLVE data, we next compared clusters on the same measures collected in previous evaluations at 5-year and 1-year follow-ups. Interestingly, at prior time points the same clusters were not well defined, suggesting a functional evolution of these key profiles over this 10-year period (Figure 2; eFigure 2 in Supplement 1). At the 1-year follow-up, a smaller subset of neurobehavioral and psychological measures were collected. All clusters were found to be significantly different when compared over time (eFigure 2 in Supplement 1). Within each primary outcome domain, neurobehavioral and psychological and cognitive, comparing 10-year with 5-year outcomes in the same cluster, 6 of 8 comparisons were also significantly different (eFigure 2 in Supplement 1). Comparing 10-year to 1-year primary outcome domains in the same cluster identified 7 of 8 significant comparisons (eFigure 2 in Supplement 1). All conclusions held in rank-based sensitivity analysis, lending further support for the evolution of these features over the first decade after deployment.

### Brain Volume Measures by Postdeployment Subtype and Over Time

At the 10-year follow-up, there were also significant brain volume differences by cluster, with MD risk–Cog having the lowest brain volume in certain regions, suggesting there may be neuroanatomical associations with these risk-resilience profiles (Table 2). Following Freesurfer volumetric segmentation for 10-year follow-up data, 7 regions remained significantly different after Benjamini-Hochberg correction (5% FDR; m = 168 regions). These included the brain stem, left and right cerebellar cortex, left cerebellar white matter, left lingual cortex and white matter, and the right cuneus. Again, leveraging the longitudinal data, we compared all volumetric regions across these clusters at 5 years, 1 year, and enrollment. In contrast to prior analyses comparing patients with TBI vs controls where no significant volumetric differences were identified,^11,18,51,52^ reexamination by these postdeployment subtypes revealed significant differences in brain volume tracking all the way back to enrollment, although clusters were based on clinical outcomes alone without reference to neuroimaging. At enrollment, there were statistically significant differences that held after correction in the left cerebellar cortex, brainstem, and very close to significance in the right cerebellar cortex (Table 2). These findings followed the same pattern observed at the 1 year, 5 years, and 10 years, with MD risk–Cog having the lowest brain volume in these regions. This general finding held at every time point, with the exception of 1-year follow-up, when these regions did not hold after strict correction (Table 2).

**Table 2.** Longitudinal Volumetric Comparisons by Postdeployment Subtype Cluster

Region	Volume, mean (SD), cm^3^				*P* value^e^
	MD-resilient^a^	Mild risk–NP^b^	MD risk–Cog^c^	MD risk–NP^d^	
**10-y Follow-up imaging**					
Left cerebellum cortex	55.9 (5.5)	58.1 (5.4)	53.2 (7.5)	55.4 (4.7)	.001
Right cerebellum cortex	56.5 (5.7)	58.8 (5.4)	53.5 (7.2)	56.3 (5.1)	.001
Brain stem	22.4 (2.1)	23.4 (2.4)	21.6 (2.7)	22.1 (2.1)	.001
Left cerebellum white matter	14.9 (2.2)	16.2 (3.2)	14.2 (3.0)	15.0 (3.2)	.001
Left lingual cortex	6.2 (1.0)	6.4 (0.9)	5.5 (0.8)	6.1 (0.9)	.002
Left lingual white matter	3.1 (0.6)	3.3 (0.5)	2.8 (0.5)	3.0 (0.4)	.001
Right cuneus	6.5 (1.0)	6.6 (0.9)	5.8 (0.7)	6.0 (0.9)	.001
**5-y Follow-up imaging**					
Left cerebellum cortex	52.4 (5.0)	55.0 (5.2)	49.5 (7.2)	52.9 (4.9)	<.001
Right cerebellum cortex	53.6 (5.2)	56.1 (5.3)	50.9 (6.7)	53.8 (5.3)	<.001
Brain stem	22.6 (2.2)	23.6 (2.5)	21.8 (2.9)	22.3 (2.1)	.001
Right cerebellum white matter	16.3 (2.0)	17.4 (2.3)	15.9 (2.0)	16.1 (2.0)	.001
Right isthmus cingulate white matter	3.6 (0.6)	3.7 (0.5)	3.1 (0.4)	3.6 (0.6)	.001
**1-y Follow-up imaging**					
Left cerebellum cortex	55.9 (5.4)	58.0 (5.2)	52.7 (7.4)	56.0 (5.2)	.007^f^
Right cerebellum cortex	57.0 (5.3)	59.2 (5.1)	53.8 (6.9)	57.0 (5.3)	.002^f^
Brain stem	23.1 (2.2)	24.0 (2.5)	22.2 (2.8)	22.5 (2.4)	.006^f^
**Baseline enrollment imaging**					
Left cerebellum cortex	55.8 (5.7)	58.0 (5.7)	52.0 (7.0)	56.3 (5.6)	.001
Right cerebellum cortex	57.6 (6.2)	59.8 (6.1)	53.7 (7.3)	58.1 (6.1)	.003^f^
Brain stem	22.2 (2.4)	23.4 (2.5)	21.3 (2.9)	21.9 (2.5)	<.001
Right thalamus	7.9 (0.9)	8.4 (1.0)	7.7 (1.3)	7.6 (0.9)	<.001
Right caudate	3.8 (0.6)	4.0 (0.6)	3.7 (0.4)	3.7 (0.6)	.001

Abbreviation: MD, multidomain.

^a^ Classified as individuals resilient to cognitive dysfunction and neurobehavioral and psychological symptoms.

^b^ Classified as individuals resilient to cognitive dysfunction but with elevated neurobehavioral and psychological symptoms.

^c^ Classified as individuals with severe cognitive dysfunction and moderately elevated neurobehavioral and psychological symptoms.

^d^ Classified as individuals with moderate cognitive dysfunction and severe neurobehavioral and psychological symptoms.

^e^ Statistical significance for omnibus cluster differences by Kruskal-Wallis without correction for multiple comparisons. Regions reported summarize those that remained statistically significant (*P* < .05) after correction for multiple comparisons (Benjamini-Hochberg, 5% FDR, m = 168).

^f^ Indicates associations that lost statistical significance after strict correction.

## Discussion

This cohort study of clinical outcome clustering at the 10-year follow-up in combat-deployed military service members found key postdeployment subtypes with corresponding brain volume differences. Further examination of these clinical features by cluster at the 5-year and 1-year follow-up waves revealed they had evolved over time. This provides important supportive evidence as to how combat exposure, including combat mild TBI, may contribute to patterns of clinical impairment over time with implications for later life neurodegeneration and dementia.^6^ Notably, while 2 of the 4 clusters were identified as having MD risk, there was a group of service members largely resilient to poor outcomes. MD-resilient did include some service members who sustained blast-related mild TBI in combat. This highlights an important complexity regarding mild TBI, underscoring the need to better appreciate not only the heterogeneity of brain injury but the associations of blast TBI with resilience and long-term outcomes.

While the neurotrauma community appreciates the concept of the miserable minority,^54^ who, and how, this subset is defined, and how best to predict who will end up in this group has largely eluded a solid operational definition. Stated more explicitly, it was encouraging to see most of the service members who had experienced blast mild TBI clustered in the more resilient trajectories. Furthermore, the consideration of cognitive reserve^55,56^ has largely been assumed to be a neuroanatomical correlate and associated with larger brain volume.^57^ In this study, we identified neuroanatomical regions, specifically the cerebellum and brainstem, that may provide greater sensitivity in identifying individuals at highest risk of decline earlier on, as these regional differences were evident years before when stratified by 10-year follow-up cluster. A growing body of literature supports the role the cerebellum plays in cognition^58,59^ and psychological health^60^ even psychological trauma^61^ relevant to combat. The brainstem has been associated with complex anatomy controlling emotion^62^ and cognition in the context of dementia.^63^ While brain structure and function is complex and not one-to-one, this prior literature does lend explanatory support for the current findings.

### Strengths and Limitations

Strengths of this study include the prospective, observational, longitudinal cohort design, with participants enrolled in combat and undergoing evaluation at 1 year, 5 years, and 10 years, with the use of a fairly comprehensive outcome battery (neurobehavioral, psychological, cognitive assessments) and high resolution MRI at all 4 time points. Additionally, we used multiple control and TBI groups for comparison with ascertainment of psychological treatment utilization^53^ tracked over time.

This study also has some limitations. Limitations include variability in care sought by the service members in the ensuing years. EVOLVE maintains participants in all 50 states, with a small number still overseas, so we were not able to confirm all treatment strategies and medications used beyond self-report. Additionally, we lacked comprehensive predeployment clinical data, and there were unknown variables that may have influenced each participant’s clinical course. Furthermore, the 2 higher-risk clusters had slightly higher rates of subsequent head injury exposure, which may have increased their risk of adverse outcomes outside of our analysis based on their index injury. While overall treatment varied, 80% of service members in the nonblast control and blast TBI groups endorsed seeking mental health care from a licensed practitioner in the ensuing years, but only 45% of nonblast controls and 30% of service members with blast mild TBI found sustained resolution of these psychological symptoms.^16^ Furthermore, possible model overfitting cannot be ruled out, and investigations into the neural mechanisms underlying these cluster trajectories were not undertaken, which are the focus of future ongoing work after validation in additional independent cohorts.

## Conclusions

The findings of this cohort study of military service members suggest that focusing on individuals whose profile most closely aligns with the MD risk–Cog and MD risk–NP clusters could provide strategic selection for risk stratification and inform intervention. Future work is needed to understand the translatability of these findings to other longitudinal TBI cohorts^64^ and the extent to which these imaging findings may be used as early biomarkers of long-term risk or resilience. Consideration of these subtypes beyond traditional exposure groups, such as control and TBI, appears warranted as research continues in this military population.

## Notes

Supplement 1. **eTable.** Summary of Cluster Modeling Fit Statistics **eFigure 1.** Summary of Cluster Effects (Z-Scores) **eFigure 2.** Summary of Year Effects Within Each Cluster (Z-scores) 
eMethods.

Supplement 2.
Data Sharing Statement
