# Supplementary material for: Long-Term Postdeployment Clinical Subtypes of Risk and Resiliency in Brain Injury and Neurodegeneration
Source: JAMA Netw Open. 2025 Dec 10;8(12):e2547954. doi: 10.1001/jamanetworkopen.2025.47954 (PMC12696601; doi:10.1001/jamanetworkopen.2025.47954)
Supplement: Supplement 1. — eTable. Summary of Cluster Modeling Fit Statistics eFigure 1. Summary of Cluster Effects (Z-Scores) eFigure 2. Summary of Year Effects Within Each Cluster (Z-scores) eMethods. [file jamanetwopen-e2547954-s001.pdf]

Supplemental Online Content

Mac Donald CL, Barber J, Hunt D, et al. Long-term post-deployment clinical subtypes of risk and resiliency in brain injury and neurodegeneration. JAMA Netw Open. 2025;8(12):e2547954. doi:10.1001/jamanetworkopen.2025.47954

eTable. Summary of Cluster Modeling Fit Statistics

eFigure 1. Summary of Cluster Effects (Z-Scores)

eFigure 2. Summary of Year Effects Within Each Cluster (Z-scores)

eMethods.

This supplemental material has been provided by the authors to give readers additional information about their work.

© 2025 Mac Donald CL et al. *JAMA Network Open*.

eTable 1 - Summary of Cluster Modeling Fit Statistics

Clusters	Pseudo F Statistic [1]	Approx. Expected Overall R-Squared (%)	Cubic Clustering Criterion [2]	Smallest Cluster Size
2	63.17	2.9	138	126
3	43.01	5.2	122	30
4	33.85	7.2	112	14
5	27.97	8.9	103	3
6	23.44	10.6	93	2
7	21.61	12.0	93	2
8	18.25	13.4	81	2
9	17.95	14.7	86	1

1. The Pseudo F statistic evaluates the ratio of between-cluster variance to within-cluster variance, with larger values indicating better separation among clusters.

2. The Cubic Clustering Criterion compares the separation of clusters in the data to what would be expected under a uniform distribution, with higher values indicating stronger evidence of meaningful clustering.

© 2025 Mac Donald CL et al. *JAMA Network Open*.

eFigure 1 - Summary of Cluster Effects (Z-Scores)

	Measure<br>Z-Scores are standardized to the overall 10yr Mean/SD	Cluster 1<br>MD Resilient	Cluster 2<br>Mild Risk<br>Neuro/Psych	Cluster 3<br>MD Risk<br>Cognitive	Cluster 4<br>MD Risk<br>Neuro/Psych
Neurobehavioral/Psychological Measures	Global Disability (GOSE)	0.73	-0.39	-0.87	-1.12
	Neurobehavior (NRS)	0.83	-0.40	-0.87	-1.49
	Headache (MIDAS)	0.43	-0.15	-0.56	-0.98
	Headache (HIT-6)	0.66	-0.35	-0.67	-1.06
	Neurological Deficits (NOS-TBI)	0.51	-0.20	-0.90	-0.95
	Quality of Life (QOL)	0.78	-0.41	-0.61	-1.38
	Pain (PROMIS Pain)	0.74	-0.38	-0.78	-1.21
	PTSD Severity (CAPS)	0.80	-0.38	-0.75	-1.52
	Self-Assessed PTSD (PCL-M)	0.84	-0.45	-0.51	-1.52
	Depression (MADRS)	0.74	-0.30	-0.50	-1.74
	Self-Assessed Depression (BDI-II)	0.76	-0.31	-0.55	-1.73
	Anxiety (BSI-A)	0.66	-0.24	-0.26	-1.73
	Sleep Difficulty (ISI)	0.75	-0.47	-0.33	-1.12
	Resilience (CD-RISC)	0.57	-0.24	-0.59	-1.18
	Alcohol Misuse (MAST)	0.20	-0.20	0.09	-0.06
	Overall Change from Cluster 1	---	B = -0.99<br>(-1.10, -0.89)<br>p < .001	B = -1.24<br>(-1.48, -1.01)<br>p < .001	B = -1.92<br>(-2.09, -1.75)<br>p < .001
	Overall Change from Cluster 2	---	---	B = -0.25<br>(-0.25, -0.02)<br>p = .032	B = -0.93<br>(-1.10, -0.76)<br>p < .001
	Overall Change from Cluster 3	---	---	---	B = -0.67<br>(-0.94, -0.41)<br>p < .001
	Omnibus Cluster Effect (p)	<.001			

	Measure<br>Z-Scores are standardized to the overall 10yr Mean/SD	Cluster 1<br>MD Resilient	Cluster 2<br>Mild Risk<br>Neuro/Psych	Cluster 3<br>MD Risk<br>Cognitive	Cluster 4<br>MD Risk<br>Neuro/Psych
Cognitive Measures	Pre-Injury Intelligence (WTAR)	0.18	0.00	-0.82	-0.40
	Attention Lapses (CPT-Omissions)	0.09	0.18	-0.39	-0.98
	Impulsivity (CPT-Comissions)	0.27	-0.12	-0.30	-0.52
	Reaction Time (CPT-Hit Rate)	-0.07	0.22	-0.24	-0.51
	Vigilence (CPT-Hit Rate Block Change)	-0.02	0.03	-0.33	0.11
	Verbal Memory (CVLT-Long Delay)	0.11	0.12	-0.98	-0.52
	False Recall (CVLT-Intrusions)	0.03	0.11	-0.45	-0.38
	Proactive Memory Interference (CVLT B vs. A)	0.08	0.01	-0.34	-0.28
	Visuospatial Learning (RULIT Total)	0.07	0.07	-0.78	-0.24
	Visuospatial Memory (RULIT Delay)	0.03	0.10	-0.52	-0.28
	Visual Scanning (Trails A)	0.23	0.13	-2.30	-0.41
	Mental Flexibility (Trails B)	0.17	0.16	-2.53	-0.19
	Verbal Fluency (COWA)	0.15	-0.01	-1.08	-0.07
	Monetary Decisions (IGT)	0.16	-0.02	-0.33	-0.45
	Naming, Reading (DKEFS, T1+T2)	0.20	0.08	-2.06	-0.21
	Inhibition (DKEFS, T3)	0.18	0.12	-2.57	-0.01
	Inhibition Switching (DKEFS, T4)	0.24	0.06	-1.89	-0.40
	Motor Speed/Coordination (GPB Average)	0.25	-0.01	-1.38	-0.35
	Motor Coordination/Strength (25ft Walk)	0.29	0.01	-0.97	-0.82
	Overall Change from Cluster 1	---	B = -0.07<br>(-0.17, 0.02)<br>p = .119	B = -1.21<br>(-1.41, -1.00)<br>p < .001	B = -0.50<br>(-0.66, -0.35)<br>p < .001
	Overall Change from Cluster 2	---	---	B = -1.13<br>(-1.34, -0.92)<br>p < .001	B = -0.43<br>(-0.58, -0.27)<br>p < .001
	Overall Change from Cluster 3	---	---	---	B = 0.70<br>(0.46, 0.95)<br>p < .001
	Omnibus Cluster Effect (p)	<.001			

© 2025 Mac Donald CL et al. *JAMA Network Open*.

Statistical significance based on mixed-effects regression with cluster, year, and cluster*year modelled as fixed effects, and subject and measure modelled as crossed random effects. Cells report the mean of each measure standardized by the overall 10yr mean/SD (Z-score transformation) and are color-coded such that blue corresponds to a good outcome and red corresponds to a bad outcome. Color shading was done as a continuous gradient representing the magnitude of the standardized effect. Effect sizes can be interpreted as the overall mean difference in the Z-score means, or equivalently as the average number of standard deviations separating the two clusters.

© 2025 Mac Donald CL et al. *JAMA Network Open*.

eFigure 2 - Summary of Year Effects Within Each Cluster (Z-scores)

	Measure (Z-Scores are standardized to the overall 10yr Mean/SD)	Cluster 1 MD Resilient			Cluster 2 Mild Risk - Neuro/Psych			Cluster 3 MD Risk - Cognitive			Cluster 4 MD Risk - Neuro/Psych		
		Y10	Y5	Y1	Y10	Y5	Y1	Y10	Y5	Y1	Y10	Y5	Y1
Neurobehavioral/Psychological Measures	GOSE	0.73	0.53	0.49	-0.39	-0.44	-0.05	-0.87	-0.75	-0.39	-1.12	-1.01	-0.39
	NRS	0.83	0.69	0.94	-0.40	-0.47	0.30	-0.87	-0.99	0.04	-1.49	-1.29	-0.24
	MIDAS	0.43	0.35	0.18	-0.15	-0.40	-0.53	-0.56	-0.93	-0.02	-0.98	-0.82	-1.02
	HIT-6	0.66	0.34	0.54	-0.35	-0.50	0.14	-0.67	-0.96	0.12	-1.06	-1.12	-0.46
	NOS	0.51	0.86	1.23	-0.20	0.45	1.14	-0.90	0.31	1.13	-0.95	-0.06	1.15
	QOL	0.78	0.68	n/a	-0.41	-0.33	n/a	-0.61	-0.96	n/a	-1.38	-0.93	n/a
	PromisPain	0.74	n/a	n/a	-0.38	n/a	n/a	-0.78	n/a	n/a	-1.21	n/a	n/a
	CAPS	0.80	0.79	0.83	-0.38	-0.45	-0.11	-0.75	-0.62	0.05	-1.52	-1.12	-0.64
	PCL-M	0.84	0.78	n/a	-0.45	-0.37	n/a	-0.51	-0.54	n/a	-1.52	-1.03	n/a
	MADRS	0.74	0.60	0.53	-0.30	-0.48	-0.26	-0.50	-0.81	-0.24	-1.74	-0.96	-0.63
	BDI-II	0.76	0.70	n/a	-0.31	-0.30	n/a	-0.55	-0.38	n/a	-1.73	-1.08	n/a
	BSI-Anxiety	0.66	0.51	n/a	-0.24	-0.34	n/a	-0.26	-0.34	n/a	-1.73	-1.15	n/a
	ISI	0.75	0.66	n/a	-0.47	-0.31	n/a	-0.33	-0.43	n/a	-1.12	-0.80	n/a
	CD-RISC	0.57	n/a	n/a	-0.24	n/a	n/a	-0.59	n/a	n/a	-1.18	n/a	n/a
	MAST	0.20	0.22	0.09	-0.20	-0.01	0.13	0.09	-0.03	0.31	-0.06	-0.05	0.25
	Overall Change from Year 10	---	B = -0.08 (-0.14, -0.03) p = .004	B = -0.06 (-0.13, 0.01) p = .001	---	B = 0.02 (-0.03, 0.08) p = .391	B = 0.35 (0.27, 0.43) p < .001	---	B = 0.00 (-0.15, 0.16) p = .952	B = 0.64 (0.43, 0.85) p < .001	---	B = 0.34 (0.23, 0.46) p = .004	B = 0.98 (0.83, 1.13) p < .001
	Overall Change from Year 5	---	---	B = 0.02 (-0.05, 0.09) p = .606	---	---	B = 0.33 (0.25, 0.41) p < .001	---	---	B = 0.64 (0.42, 0.85) p < .001	---	---	B = 0.64 (0.48, 0.79) p < .001
	Year Effect (p)	.001			<.001			<.001			<.001		
	Difference in Year Effects	<.001											

	Measure (Z-Scores are standardized to the overall 10yr Mean/SD)	Cluster 1 MD Resilient			Cluster 2 Mild Risk - Neuro/Psych			Cluster 3 MD Risk - Cognitive			Cluster 4 MD Risk - Neuro/Psych		
		Y10	Y5	Y1	Y10	Y5	Y1	Y10	Y5	Y1	Y10	Y5	Y1
Cognitive Measures	Wechsler	0.18	0.01	-0.35	0.00	-0.11	-0.51	-0.82	-0.87	-1.30	-0.40	-0.69	-1.15
	Conners Omission	0.09	0.12	-0.27	0.18	0.11	-0.91	-0.39	-0.28	-1.45	-0.98	-0.29	-1.42
	Conners Commission	0.27	0.34	0.06	-0.12	-0.11	-0.23	-0.30	-0.45	-0.37	-0.52	-0.40	-0.96
	Conners Hit Rate	-0.07	-0.05	0.13	0.22	0.12	0.57	-0.24	0.26	0.54	-0.51	-0.07	0.57
	Conners Hit Rate Block	-0.02	-0.05	0.15	0.03	0.08	0.05	-0.33	-0.04	-0.04	0.11	0.02	-0.27
	CVLT Long Delay	0.11	0.11	-0.13	0.12	0.00	-0.18	-0.98	-0.71	-0.54	-0.52	-0.31	-0.45
	CVLT Total Intrusions	0.03	0.34	0.01	0.11	0.28	-0.07	-0.45	-0.27	-0.03	-0.38	0.05	-0.31
	CVLT B vs A	0.08	0.27	0.17	0.01	0.21	0.14	-0.34	0.45	0.22	-0.28	-0.16	0.32
	Ruff Total	0.07	0.10	0.05	0.07	0.03	-0.08	-0.78	-0.61	-0.18	-0.24	-0.23	0.13
	Ruff Long Delay	0.03	0.12	0.17	0.10	0.02	0.09	-0.52	-0.88	-0.33	-0.28	-0.12	-0.13
	Trails A	0.23	0.45	0.36	0.13	0.35	0.16	-2.30	-0.72	-0.36	-0.41	-0.13	-0.33
	Trails B	0.17	0.37	0.33	0.16	0.25	0.27	-2.53	-0.39	-0.28	-0.19	-0.14	0.14
	COWAT	0.15	0.21	-0.18	-0.01	0.17	-0.23	-1.08	-0.65	-0.94	-0.07	-0.02	-0.06
	Iowa Gambling	0.16	0.25	0.04	-0.02	0.10	0.18	-0.33	-0.14	-0.34	-0.45	-0.63	-0.13
	D-KEFS Trial 1+2	0.20	0.40	0.54	0.08	0.44	0.47	-2.06	-0.87	0.04	-0.21	0.17	0.21
	D-KEFS Trial 3	0.18	0.23	0.10	0.12	0.01	-0.01	-2.57	-1.24	-0.86	-0.01	-0.15	-0.35
	D-KEFS Trial 4	0.24	0.24	0.02	0.06	0.01	0.02	-1.89	-1.13	-0.48	-0.40	-0.33	-0.51
	Grooved Pegboard Avg	0.25	0.45	0.21	-0.01	0.26	0.02	-1.38	-0.31	-0.40	-0.35	-0.06	-0.16
	25ft Walk	0.29	0.70	0.67	0.01	0.48	0.36	-0.97	0.08	0.19	-0.82	0.22	0.30
	Overall Change from Year 10	---	B = 0.10 (0.04, 0.16) p = .001	B = -0.07 (-0.13, 0.00) p = .040	---	B = 0.07 (0.01, 0.13) p = .024	B = -0.09 (-0.15, -0.02) p = .010	---	B = 0.60 (0.44, 0.77) p < .001	B = 0.66 (0.47, 0.84) p < .001	---	B = 0.20 (0.08, 0.32) p = .001	B = 0.11 (-0.02, 0.24) p = .096
	Overall Change from Year 5	---	---	B = -0.16 (-0.23, -0.10) p < .001	---	---	B = -0.15 (-0.22, -0.09) p < .001	---	---	B = 0.05 (-0.13, 0.24) p = .571	---	---	B = -0.09 (-0.22, 0.05) p = .194
	Year Effect (p)	<.001			<.001			<.001			.019		
	Difference in Year Effects	<.001											

© 2025 Mac Donald CL et al. *JAMA Network Open*.

n/a = not administered
Statistical significance based on mixed-effects regression with cluster, year, and cluster*year modelled as fixed effects, and subject and measure modelled as crossed random effects. Cells report the mean of each measure standardized by the overall 10yr mean/SD (Z-score transformation) and are color-coded such that blue corresponds to a good outcome and red corresponds to a bad outcome. Color shading was done as a continuous gradient representing the magnitude of the standardized effect. Effect sizes can be interpreted as the overall mean difference in the Z-score means, or equivalently as the average number of standard deviations separating the two time cohorts.

© 2025 Mac Donald CL et al. *JAMA Network Open*.

1. eMethods

Service members underwent MRI scanning at all time points including enrollment and assessed with a comprehensive battery of neurobehavioral, psychological, and cognitive test measures at each follow-up time point (1-year, 5-year, 10-year post-injury or enrollment). Some data missingness was identified and the primary reason for missing data was the cognitive assessment battery and MRI scan that could only be performed in person which excluded individuals who completed remote follow up. As the main focus of this cluster analysis was to understand long-term post-deployment subtypes across domains of function including neurobehavioral, psychological and cognitive, and possible brain imaging correlates this exclusion was felt to be justified.

1.1 Clinical Outcomes Evaluations

All tests were performed between 8 am and 5 pm in private, quiet, well-lit rooms. Per patient, the examiners for each evaluation battery were different, meaning the patient would see three different examiners for the three different assessments (neurobehavioral, psychological, cognitive). To evaluate multiple patients on a single day, assessment order for the neurobehavioral and psychological evaluations varied making sure to always complete the cognitive assessment in the first half of the day.

1.2 Glasgow Outcome Scale Extended (GOSE) Scoring

The GOSE is scored from 1-8: 1=dead, 2=vegetative, 3-4=severe disability, 5-6=moderate disability, 7-8=good recovery. Moderate disability (GOSE = 5-6) is defined as one or more of the following: 1) inability to work to previous capacity 2) inability to resume much of regular social and leisure activities outside the home 3) psychological problems which have frequently resulted in ongoing family disruption or disruption of friendships. Severe disability (GOSE = 3-4) is defined as one or more of the following: 1) inability to drive and/or travel locally without assistance 2) inability to shop or run errands without assistance 3) support required for activities of daily living. Standardized, structured interviews were performed per published guidelines[1]. Participants were instructed to consider deployment as the reference point for this inquiry.

© 2025 Mac Donald CL et al. *JAMA Network Open*.

1.3 Clinician Administered PTSD Scale (CAPS) Scoring

The CAPS was scored using the standards from Blake et al[2]. The DSM-IV version of CAPS was used at all follow-up evaluation waves as the study started in 2008 when this was the standard and it was collectively agreed by the study clinicians that it would be best to maintain the version for more direct comparison than switch to the DSM-V[3] version when it came out in 2013.

1.4 Cognitive Assessment Details

The following cognitive measures were completed at each follow-up wave: Wechsler Test of Adult Reading (WTAR) [4], an estimate of pre-injury verbal intelligence reported as a Standard Score; Conner's Continuous Performance Test II [5](CPT), a computer-based assessment of attention lapses(CPT-Omissions), impulsivity (CPT-Commissions), reaction time (CPT-Hit Rate), and vigilance (CPT-Hit Rate Block Change) all reported as T-Scores; California Verbal Learning Test II [6](CVLT), an assessment of verbal declarative memory (CVLT-Long Delay), falsely recalled items (CVLT-Intrusions), and proactive memory interference (CVLT B vs. A) all reported as Standard Score; Ruff-Light Trail Learning Test[7], an assessment of visuospatial learning (RULIT Total) reported as T-Score and visuospatial memory (RULIT Delay) reported as Raw Score (15 max); Trail Making Test, an assessment of visual scanning (Trails A) and mental flexibility (Trails B) [8] both reported as time in seconds; Controlled Oral Word Association Test (COWA) [9] reported as Total Score, an assessment of verbal fluency ; Iowa Gambling Test(IGT)[10], a computer-based assessment of impulsivity and monetary decision making reported as T-Score; Delis-Kaplan Executive Function System (D-KEFS) Color-Word Interference Test [11], an multi-domain assessment of executive function including naming/reading (DKEFS T1+T2), inhibition (DKEFS T3) and inhibition/switching (DKEFS T4) all reported as Scaled Score. Fine motor and gross motor function were assessed by the 25-hole Grooved Pegboard Test [12], an assessment of upper extremity motor speed and coordination (GPB average) and the timed 25-foot walk, an assessment of motor strength, balance, coordination (25ft Walk) both reported as time in seconds. Embedded metrics from CVLT

© 2025 Mac Donald CL et al. *JAMA Network Open*.

(forced choice) assessed adequacy of effort and were done as far apart as possible from other verbal measures to reduce verbal learning interference. For more information regarding the details of the EVOLVE cognitive testing data over time, please see our prior published work[13,14].

1.5 Quantitative Brain Volume Analysis Methods

FreeSurfer is a semi-automatic segmentation program for analyzing volumetric data using a high resolution structural T1-weighted image. This process is divided into two primary parts. The first part consists of sub-cortical/white matter surface creation and segmentation of the individual structures. The second part provides reconstruction of the cortical surface, created from the underlying white matter surface followed by parcellation of the cortical areas. Image processing steps include initial regression of extraneous, non-cortex/non-white matter tissue and motion correction before alignment with the MNI template. Spatial registration of the brain mask to the MNI space is completed prior to the elimination of topological defects. After this, the generation of the 'pial/cortical' and 'white matter' tessellated surfaces is completed. Quality assurance of the image processing includes review of QA/QC measures including noise values, null values, fit measures. Additionally, complete visual inspection in each orthogonal plane of the segmentation is done with application of control points where needed to assure correct alignment of the automated FreeSurfer program and to correct for small erroneous inclusions of other anatomy such as blood vessels, dura, and any white matter lesions. This is completed by an imaging scientist blinded to the clinical status of the EVOLVE service member, who has been trained in neuroanatomy and is an expert using the FreeSurfer processing software.

SUPPLEMENTAL REFERENCES

1. Wilson JT, Pettigrew LE, Teasdale GM. Structured interviews for the Glasgow Outcome Scale and the extended Glasgow Outcome Scale: guidelines for their use. *J Neurotrauma*. 1998;15(8):573-585.
2. Blake DD, Weathers FW, Nagy LM, et al. The development of a Clinician-Administered PTSD Scale. *J Trauma Stress*. 1995;8(1):75-90.
3. Weathers FW, Blake DD, Schnurr PP, Kaloupek DG, Marx BP, Keane TM. The Clinician-Administered PTSD Scale for DSM-5 (CAPS-5). *National Center for PTSD* 2013.

© 2025 Mac Donald CL et al. *JAMA Network Open*.

4. Wechsler D. *Wechsler Test of Adult Reading (WTAR) Manual.* New York: Psychological Corporation; 2001.
5. Conners C, Staff. M. *Conners' Continuous Performance Test II: Computer program for Windows technical guide and software manual.* North Tonwanda, NY: Multi-Health Systems; 2000.
6. Delis D, Kramer J, Kaplan E, B O. *California Verbal Learning Test Manual: Second Edition, Adult Version.* San Antonio, Tx: Psychological Corporation; 2000.
7. Ruff R, Light R, Parker S. Visuospatial learning: Ruff Light Trail Learning Test. *Arch Clin Neuropsychol.* 1996;11(4):313-327.
8. Reitan R. *Trail Making Test manual for administration and scoring.* Tuscon, AZ: Reitan Neuropsychology Laboratory; 1992.
9. Benton A, Hamsher K, A S. *Multilingual Aphasia Examination (3rd ed.).* Iowa City, Ia: AJA Associates; 1983.
10. Bechara A, Damasio AR, Damasio H, Anderson SW. Insensitivity to future consequences following damage to human prefrontal cortex. *Cognition.* 1994;50(1-3):7-15.
11. Delis DC, Kaplan, E. & Kramer, J.H. *Delis-Kaplan Executive Function System (D-KEFS): Examiner's manual.* San Antonio, TX: The Psychological Corporation; 2001.
12. Matthews C, Kløve H. *Instruction manual for the Adult Neuropsychology Test Battery.* Madison, WI: University of Wisconsin Medical School; 1964.
13. Mac Donald CL, Barber J, Jordan M, et al. Early Clinical Predictors of 5-Year Outcome After Concussive Blast Traumatic Brain Injury. *JAMA Neurol.* 2017;74(7):821-829.
14. Mac Donald CL, Johnson AM, Wierzechowski L, et al. Outcome Trends after US Military Concussive Traumatic Brain Injury. *J Neurotrauma.* 2017;34(14):2206-2219.

© 2025 Mac Donald CL et al. *JAMA Network Open*.
