# Supplementary material for: Long-Term Postdeployment Clinical Subtypes of Risk and Resiliency in Brain Injury and Neurodegeneration
Source: JAMA Netw Open. 2025 Dec 10;8(12):e2547954. doi: 10.1001/jamanetworkopen.2025.47954 (PMC12696601; doi:10.1001/jamanetworkopen.2025.47954)
Supplement: Supplement 2. — Data Sharing Statement [file jamanetwopen-e2547954-s002.pdf]

Data Sharing Statement

Mac Donald. Long-Term Postdeployment Clinical Subtypes of Risk and Resiliency in Brain Injury and Neurodegeneration. *JAMA Netw Open*. Published December 10, 2025. doi:10.1001/jamanetworkopen.2025.47954

Data
Data available: Yes
Data types: Deidentified participant data
How to access data: Data sharing will be made available to interested parties through data use agreements with the corresponding author.
When available: With publication

Supporting Documents
Document types: Statistical/analytic code
How to access documents: Statistical/analytic code will be made available to interested parties by contacting the corresponding author. cmacd@uw.edu
When available: With publication

Additional Information
Who can access the data: Data will be made available following approved data use agreements.
Types of analyses: Data will be made available for purposes specified in the data use proposals.
Mechanisms of data availability: Data will be made available after approval of the data use agreement proposal.
Any additional restrictions: N/A
